# Antibody–drug conjugates and immunogenic cell death in breast cancer: agent-specific mechanisms, clinical evidence and translational priorities

**DOI:** 10.3389/fimmu.2026.1978134

**Published:** 2026-10-22

**Authors:** Linghui Fang, Jialu Song, Xiaohui Wang, Fan Wang, Xiaotao Zhu

**Affiliations:** Department of Breast and Thyroid Surgery, Affiliated Jinhua Hospital, Zhejiang University School of Medicine, Jinhua, Zhejiang, China

**Keywords:** antibody–drug conjugates, breast cancer, immune-checkpoint inhibitors, immunogenic cell death, tumour immune microenvironment

## Abstract

Antibody–drug conjugates (ADCs) and immune-checkpoint inhibitors (ICIs) are reshaping breast cancer treatment, but the extent to which individual ADCs induce immunogenic cell death (ICD) remains construct specific. Microtubule-inhibitor and topoisomerase I-inhibitor payloads can generate distinct forms of cellular stress and ICD-associated signals, including calreticulin (CALR) exposure, adenosine triphosphate (ATP) and high-mobility group box 1 (HMGB1) release, and type I interferon signalling. These molecular hallmarks indicate immunogenic potential but do not establish functional ICD without evidence of antigen-specific T-cell priming, immune-dependent tumour control or protective memory. Fc gamma receptor (FcγR)-mediated phagocytosis may facilitate antigen processing, whereas bystander killing primarily reflects payload diffusion and is not intrinsically immunostimulatory. Clinically, sacituzumab govitecan plus pembrolizumab improves progression-free survival over chemotherapy plus pembrolizumab in previously untreated programmed death-ligand 1 (PD-L1)-positive advanced triple-negative breast cancer, establishing the superiority of that treatment strategy without proving biological synergy or ICD-mediated benefit. This review integrates agent-specific preclinical evidence, breast cancer immune contexts, clinical trial data, resistance mechanisms and safety considerations. We propose an ADC–ICD competence framework comprising accessibility, danger signalling, immune reception and immune braking. The framework is intended to organise dynamic biomarker development and prospective validation, not to provide a numerical score or clinical decision rule.

## 1 Introduction

GLOBOCAN 2024 estimates approximately 2.4 million new cases of breast cancer in women and 694,000 deaths worldwide, confirming breast cancer as the most commonly diagnosed malignancy in women (1). In the precision-therapy era, drug development is moving beyond discrete molecular subtypes towards continuous antigen-expression spectra, spatial heterogeneity, evolving resistance and the tumour microenvironment. Bibliometric analyses identify antibody–drug conjugates (ADCs), trophoblast cell-surface antigen 2 (TROP2), human epidermal growth factor receptor 2 (HER2)-low disease, immunotherapy and clinical trials as rapidly expanding themes in breast cancer, particularly in triple-negative breast cancer (2–4).

The convergence of ADC therapy and immunotherapy is compelling for three reasons. First, ADCs have moved rapidly from late-line monotherapy into earlier treatment settings. Trastuzumab deruxtecan (T-DXd), sacituzumab govitecan (SG) and datopotamab deruxtecan (Dato-DXd) have expanded treatment options across HER2-positive, HER2-low and HER2-ultralow, hormone receptor (HR)-positive/HER2-negative, and triple-negative breast cancer (TNBC) (5–11). Second, the antibody backbone, linker, payload and drug-to-antibody ratio can each influence pharmacology and immune-cell engagement. Third, a completed randomised phase III trial supports sacituzumab govitecan plus pembrolizumab in programmed death-ligand 1 (PD-L1)-positive advanced TNBC (12). Other phase III programmes, including TROPION-Breast03 and OptimICE-RD, had not reported efficacy results at the review cutoff (13, 14). The central questions are therefore which ADC constructs generate immunologically meaningful tumour-cell injury, which tumour and host contexts can propagate those signals, and when an immune-checkpoint inhibitor (ICI) adds benefit.

Accordingly, this mechanism-oriented narrative review first examines construct- and agent-specific determinants of immune activity, then distinguishes molecular ICD-associated hallmarks from functional immune evidence. It subsequently evaluates preclinical and clinical evidence by ADC and breast cancer context, identifies resistance and safety constraints, and proposes a biomarker-development framework. The literature cutoff was 31 July 2026.

The conceptual contribution of this review is to connect ADC engineering with the successive biological requirements for immune conversion while preserving the boundaries of the underlying evidence. Tumour entry, antigen engagement, linker cleavage, payload diffusion, phagocytic processing, dendritic-cell function, T-cell priming and adaptive suppression may each influence outcome. These processes should not be treated as a universal sequence shared by all ADCs. Instead, their relevance depends on the target, antibody scaffold, linker, payload, disease subtype and experimental system.

Here, *in situ* vaccination is used as a hypothesis describing the conversion of treatment-released tumour antigens into antigen-specific adaptive immunity. The term does not imply that ADCs function as established cancer vaccines. Evidence of calreticulin exposure or damage-associated molecular pattern (DAMP) release alone is insufficient; the hypothesis requires antigen processing, T-cell priming and, ideally, immune-dependent protection or memory.

### 1.1 Scope and principles for interpreting evidence

This review is narrative rather than systematic. Clinical efficacy conclusions were prioritised according to randomised trials; paired human specimens were used to assess pharmacodynamic associations; immunocompetent models were used to evaluate immune dependence; and DAMP measurements in cell systems were interpreted as ICD-associated hallmarks. Mechanistic relationships are described as associative or supportive unless interventional evidence establishes pathway dependence.

### 1.2 Literature search and study selection

Relevant literature was identified through structured searches of PubMed/MEDLINE and ClinicalTrials.gov from database inception to 31 July 2026. Search concepts combined breast cancer with antibody–drug conjugate, trastuzumab emtansine (T-DM1), T-DXd, sacituzumab govitecan, datopotamab deruxtecan, immunogenic cell death, calreticulin, ATP, HMGB1, cyclic GMP–AMP synthase (cGAS)–stimulator of interferon genes (STING) signalling, dendritic cell, T-cell response, immune-checkpoint inhibition, resistance, pharmacokinetics and interstitial lung disease. Peer-reviewed primary studies and randomised trials were prioritised. Conference reports were considered only when they provided recent trial information not yet available in a full publication, and ongoing studies were identified from trial registries. Reference lists of key consensus statements and reviews were screened for additional mechanistic studies. Studies were selected when they directly informed ADC construct biology, functional ICD assessment, clinical efficacy, safety or biomarker development; no formal risk-of-bias assessment was undertaken.

## 2 ADC architecture and agent-specific determinants of immune activity

A classical ADC comprises an antibody, a linker and a payload. After antigen binding, many ADCs undergo endocytosis and lysosomal processing, although extracellular cleavage can contribute for selected linker–payload systems (15, 16). The released payload may injure target cells and, when sufficiently membrane permeable, neighbouring cells (17). Antigen expression alone therefore does not capture construct-specific differences in fragment crystallisable (Fc) activity (16), linker-dependent release (16), or intratumour distribution (18, 19). **Figure 1** summarises these distinct spatial and immune consequences without implying that they occur to the same extent for every ADC.

**Figure 1:**
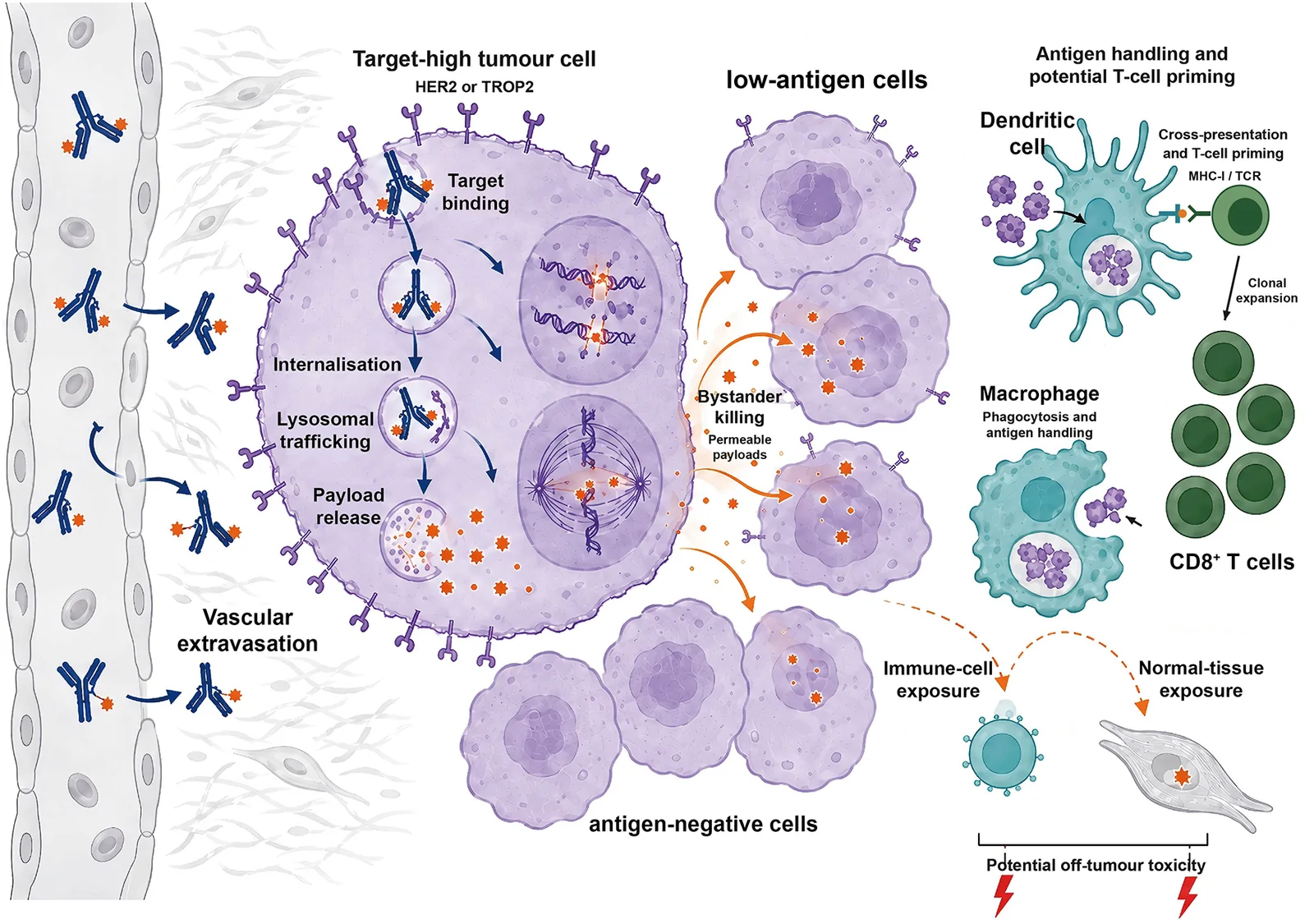
Spatial pharmacology and potential immune consequences of ADC delivery in breast cancer. Following vascular extravasation, an ADC may bind HER2- or TROP2-high tumour cells, undergo internalisation and lysosomal trafficking, and release its payload. Membrane-permeable payloads can produce bystander killing in neighbouring low-antigen or antigen-negative cells; this distributional effect is distinct from FcγR-mediated phagocytosis and is not intrinsically immunostimulatory. Tumour material may be captured by dendritic cells and macrophages. The dendritic-cell branch depicts antigen cross-presentation on major histocompatibility complex (MHC) class I and recognition by the T-cell receptor (TCR), supporting potential CD8^+^ T-cell priming and clonal expansion. The macrophage branch depicts phagocytosis and antigen handling. Off-tumour payload exposure to immune or normal-tissue compartments may contribute to toxicity. The schematic depicts potential, construct-dependent mechanisms rather than a validated sequence shared by all ADCs.

### 2.1 The antibody scaffold: from receptor blockade to Fc-gamma-receptor-mediated immune cooperation

An immunoglobulin G1 (IgG1) scaffold with an accessible Fc region can engage Fc gamma receptors (FcγRs) on natural killer cells and macrophages and thereby support antibody-dependent cellular cytotoxicity or phagocytosis. The trastuzumab scaffold of T-DXd also retains HER2-directed signalling effects. In HER2-positive preclinical models, T-DXd promoted FcγR-dependent macrophage phagocytosis (16). FcγR composition, effector-cell density and the balance of activating and inhibitory FcγRs should therefore be regarded as candidate modifiers requiring prospective validation, not as established clinical predictors.

### 2.2 Linkers and extracellular proteases: determining where the payload is released

Cleavable linkers can enable bystander killing, but release location is construct specific. Tsao et al. showed that extracellular cathepsin L (CTSL)-dependent cleavage contributed to T-DXd activity in selected HER2-low and HER2-negative preclinical models (16). This finding supports an author-proposed antigen–protease–stroma hypothesis for T-DXd, in which target distribution, extracellular cleavage and tissue penetration jointly influence local payload exposure. CTSL has not been prospectively validated as a clinical predictor of T-DXd response and should not be treated as a generic accessibility biomarker for ADCs with different linker chemistries (15–17).

### 2.3 Payload class: determining the mode of death and its immunological quality

Microtubule-inhibitor payloads can produce mitotic stress and ICD-associated signalling, as shown for the maytansinoid payload DM1 in HER2-positive breast cancer models (20). Topoisomerase I-inhibitor payloads produce DNA damage (15); their downstream immune effects depend on the construct and cellular context (16, 21). These observations do not establish a common ICD mechanism across ADCs. Drug-to-antibody ratio, payload permeability and release kinetics also influence bystander killing and exposure of neighbouring cells (15, 17).

### 2.4 Tissue distribution and the bystander effect: from single-cell targeting to spatial pharmacology

Spatial imaging of HER2-directed ADCs shows heterogeneous intratumour distribution and a perivascular binding-site barrier (19). Experiments with anti-HER2 antibodies likewise show that affinity and internalisation influence penetration beyond tumour vessels (18). Consequently, a mean HER2 or TROP2 immunohistochemistry score cannot by itself describe the exposure experienced by individual tumour and immune cells. The term local drug niche is used here as an author-proposed spatial concept for the relationship among vessels, antigen-positive cells, stromal features and immune cells.

Membrane-permeable payloads can diffuse from antigen-positive cells into adjacent low-antigen cells, thereby reducing escape driven by antigen heterogeneity (17, 19). This bystander effect primarily describes payload distribution and is not intrinsically immunostimulatory. Limited diffusion may spare antigen-negative clones, whereas broader exposure may also injure proliferating immune cells or normal tissues; these competing effects require construct- and tissue-specific measurement rather than assuming that unrestricted diffusion is desirable.

### 2.5 Exposure and time: an immune-activation window

ADC pharmacokinetics cannot be represented by a single exposure measure. Depending on the construct and development question, bioanalysis may quantify total antibody, conjugated ADC, free or conjugated payload, linker-related species and drug-to-antibody-ratio-related measurements (22). These analytes describe different aspects of target-directed delivery, deconjugation and systemic payload exposure. An exposure–toxicity relationship should therefore be established for the specific agent and analyte rather than inferred from free payload alone.

ICD-associated signals are temporally ordered: CALR exposure may precede membrane disruption, ATP release depends partly on autophagic flux, and HMGB1 is generally released later. Type I interferons and chemokines can subsequently alter immune-cell recruitment (23–25). This sequence supports pharmacodynamically timed sampling, but an optimal interval has not been clinically established. Sampling at 24–72 h after the first dose should therefore be treated as a testable window-study hypothesis rather than a validated schedule.

## 3 Defining ADC-associated ICD: molecular hallmarks, functional validation and systemic immunity

ICD is not defined by a fixed morphological form of cell death, but by a functional state in which antigenic and adjuvant signals are released in an appropriate spatiotemporal sequence and can ultimately support adaptive immune memory (23). Detection of calreticulin, ATP or HMGB1 alone establishes only the presence of ICD-related markers, not complete ICD. Rigorous confirmation additionally requires antigen-specific T-cell responses, immune-dependent tumour control and, ideally, vaccination/rechallenge experiments in immunocompetent animals (23–32).

### 3.1 The eat-me signal: calreticulin exposure

Endoplasmic-reticulum stress and eukaryotic translation initiation factor 2α (eIF2α) phosphorylation can promote cell-surface exposure of calreticulin (CALR). Surface CALR functions as an eat-me signal, enhancing recognition and phagocytosis of dying tumour cells by dendritic cells and macrophages. High tumour expression of CD47, a don’t-eat-me signal, can counteract CALR-mediated phagocytosis and act as an adaptive brake on immunity after ICD-related cell death (28, 29, 33).

### 3.2 The recruitment signal: the ATP–P2RX7–NLRP3 axis

Autophagy-dependent ATP secretion recruits myeloid cells and activates the purinergic P2X7 receptor (P2RX7) and NOD-like receptor family pyrin domain-containing 3 (NLRP3) inflammasome, promoting interleukin-1β (IL-1β) production and effector T-cell priming (34). ATP is rapidly converted to adenosine by CD39 and CD73; high tumour expression of these enzymes may shift transient immune activation towards adenosine-mediated suppression (34). Plasma ATP alone is unlikely to reflect this local biology. Tumour-interstitial enzyme measurements, myeloid-cell states and paired spatial analyses would provide a more specific test.

### 3.3 The maturation signal: the HMGB1–TLR4 axis

HMGB1 released by dying tumour cells can engage Toll-like receptor 4 (TLR4) on dendritic cells and facilitate antigen processing and cross-presentation in chemotherapy and radiotherapy models (35). Its redox state, timing and tissue concentration may alter these effects, so an isolated increase in HMGB1 does not establish effective immune priming (23, 27). Dendritic-cell maturation, antigen-specific T-cell responses and immune dependence provide stronger functional readouts.

### 3.4 The broadcast signal: cytosolic DNA, cGAS–STING and type I interferon

Experiments with unconjugated topoisomerase I poisons show that DNA damage can induce cGAS–STING-dependent immune gene expression, although this response varies across tumour-cell contexts (21). Cytosolic DNA sensing can activate cGAS and STING, with downstream TANK-binding kinase 1 (TBK1), interferon regulatory factor 3 (IRF3), type I interferon and C-X-C motif chemokine ligand 10 (CXCL10) signalling (36, 37). Separately, preclinical T-DXd studies suggest that DXd can induce ICD-related signals and enhance myeloid-cell activation and antigen presentation through TLR4 and STING (16). Pathway activation alone does not establish productive T-cell priming or functional ICD (23, 24).

### 3.5 Three requirements for ICD: antigenicity, adjuvanticity and a receptive immune context

Functional ICD requires antigenicity, adjuvanticity and a receptive immune context. Dying cells must supply tumour antigens that can be processed and recognised by T cells; CALR, ATP, HMGB1, type I interferon and related signals must be emitted in a biologically effective sequence; and dendritic-cell, lymphatic and T-cell functions must permit antigen-specific priming (23, 24). Myelosuppression, deficiency of type 1 conventional dendritic cells (cDC1) or impaired antigen-presentation pathways can uncouple DAMP release from productive adaptive immunity (38, 39).

Reductionist cell systems can demonstrate cellular stress and DAMP release but cannot reproduce tissue distribution, phagocytic competition, lymph-node migration or T-cell clonal selection. Conversely, tumour shrinkage in patients may reflect direct payload cytotoxicity without establishing ICD. We therefore apply the Level 0–4 validation framework proposed by Boaru et al. (24) to ADC-associated evidence: Level 0 denotes molecular hallmarks; Level 1, innate immune engagement; Level 2, antigen-specific T-cell priming; Level 3, evidence of immune-dependent tumour control; and Level 4, convergent human evidence. Protective memory requires appropriately controlled vaccination or rechallenge experiments. Level 4 adds human relevance but does not supersede the causal strength of Level 3 functional experiments. The adapted hierarchy and its inference boundaries are presented in **Table 1**.

**Table 1:** Evidence hierarchy for evaluating ADC-associated immunogenic cell death, adapted to the ADC context from Ref (24).

Evidence level	Representative evidence	Inference supported	Key limitation
Level 0: molecular hallmarks	CALR exposure; ATP/HMGB1 release; p-eIF2α; type I interferon-related signals	The ADC or payload has immunogenic potential	Does not demonstrate immune function, antigen specificity or ICD
Level 1: innate immune engagement	Phagocytosis; dendritic-cell maturation; cytokine production; *in vitro* cross-presentation	Dying-cell signals are biologically perceived by innate immunity	Innate activation may not generate antigen-specific T-cell responses
Level 2: adaptive priming	MHC-I-dependent priming; antigen-specific CD8^+^ T-cell proliferation or function	Treatment-associated cell death initiates adaptive immune priming	Does not establish durable protection or memory
Level 3: evidence of immune-dependent tumour control	Vaccination/rechallenge protection; CD8^+^ T-cell, Batf3/cDC1 or pathway-dependence experiments	Functional evidence of immune-dependent tumour control; protective memory requires appropriately controlled vaccination or rechallenge experiments.	Model-dependent and not sufficient to establish clinical relevance
Level 4: human relevance	Paired tissues; T-cell repertoire dynamics; spatial immune changes; associations with outcome	Human pharmacodynamic relevance and clinical plausibility	Usually correlative; does not establish causality or treatment synergy

Adapted to the ADC context from Boaru et al. (24), under the Creative Commons Attribution 4.0 International licence (https://creativecommons.org/licenses/by/4.0/). Source: https://doi.org/10.3390/ijms27073061.

### 3.6 From local inflammation to systemic immunity: the underappreciated role of draining lymph nodes

Tumour-cell death *in situ* is not equivalent to systemic immunity. Antigen capture and cross-presentation by dendritic cells must be followed by T-cell priming in lymphoid tissue before expanded clones can contribute to responses at primary or distant sites (23, 38). In an immunocompetent TNBC model, tumour-draining lymph nodes supported priming against lymphatically transported tumour antigen, illustrating the importance of this compartment without establishing that ADC treatment engages the same pathway (40). Sampling a single biopsied lesion cannot by itself distinguish local bystander killing from systemic immune effects.

Clinical studies can integrate multiplex tissue imaging with peripheral-blood T-cell receptor (TCR) sequencing, circulating biomarkers and circulating tumour DNA (ctDNA) dynamics. A temporal sequence linking tumour DAMPs and cDC1 activation to subsequent expansion of tumour-reactive TCR clones and regression of non-biopsied lesions would strengthen, but not prove, an *in situ* vaccination hypothesis. Inflammatory mediators without antigen-specific clonal expansion may instead reflect nonspecific tissue injury. **Figure 2** summarises this conceptual pathway.

**Figure 2:**
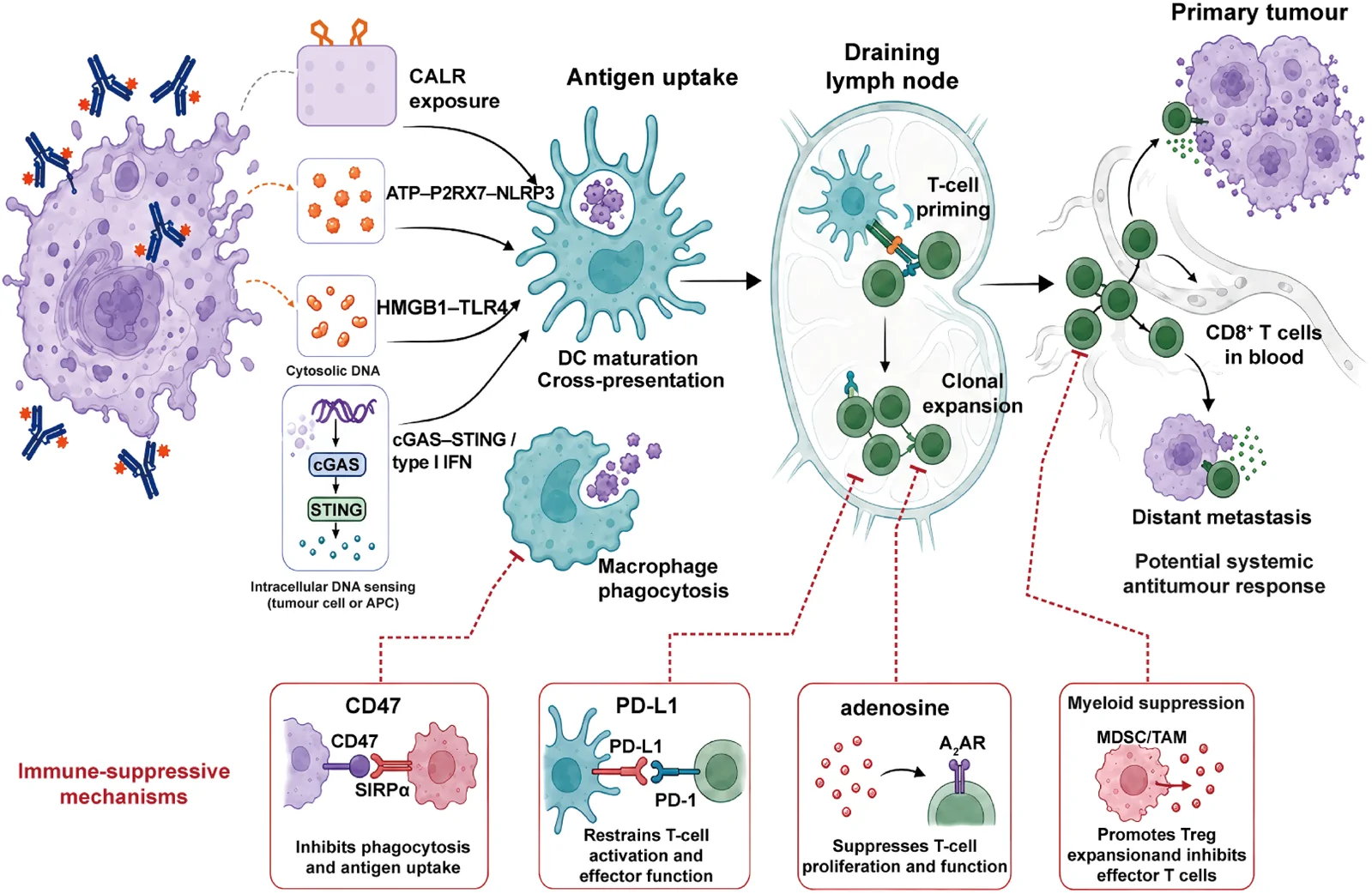
Potential pathway from ADC-induced ICD-associated signalling to systemic antitumour immunity. ADC-treated tumour cells may expose calreticulin (CALR) and release ATP, HMGB1 and tumour antigens. These signals can promote antigen uptake, dendritic-cell maturation and cross-presentation. The labelled ATP–P2RX7–NLRP3 and HMGB1–TLR4 axes depict candidate routes of innate immune signalling. Cytosolic DNA sensing in tumour cells or antigen-presenting cells may activate cGAS–STING and type I interferon signalling. In the draining lymph node, antigen presentation can support CD8^+^ T-cell priming and clonal expansion, followed by trafficking to primary and distant tumour sites. Macrophage phagocytosis may additionally contribute to antigen handling. CD47–signal regulatory protein α (SIRPα) signalling, programmed cell death protein 1 (PD-1)–PD-L1 signalling, adenosine–adenosine A_2_A receptor (A_2_AR) signalling and myeloid suppression are depicted as immune-suppressive mechanisms that can interrupt this pathway. Dashed red lines ending in T-bars denote inhibition of the indicated immune processes. The scheme is conceptual; detection of damage-associated molecular patterns (DAMPs) or clinical activity alone does not establish durable systemic immune memory or ICD-mediated clinical synergy.

## 4 Agent-specific preclinical evidence in breast cancer

### 4.1 T-DM1: mitotic arrest and ICD-related signalling induced by a microtubule payload

Trastuzumab emtansine (T-DM1) has established clinical efficacy in HER2-positive advanced breast cancer and in patients with residual invasive disease after neoadjuvant therapy (41, 42). *In vitro* studies have shown that maytansinoid ADCs can induce CALR exposure, ATP secretion and HMGB1 release selectively in antigen-positive cells; these findings constitute molecular hallmarks rather than functional proof of ICD (43). In HER2-positive breast cancer models, T-DM1-associated DM1 exposure has been linked to spindle-assembly-checkpoint-dependent mitotic arrest, eIF2α phosphorylation and ICD-associated signalling (20). The same study reported attenuation of these signals in resistant cells, response-associated signatures and post-treatment CD8^+^ T-cell infiltration (20). It does not establish that resistant cells retain equivalent killing while selectively losing immunogenicity; the interpretation is therefore limited to attenuation of ICD-associated signalling.

### 4.2 T-DXd: extracellular release, Fc-gamma-receptor-dependent phagocytosis and STING activation

T-DXd combines a high drug-to-antibody ratio, a cleavable linker and a membrane-permeable DXd payload. Its clinical activity extends across HER2-positive, HER2-low and HER2-ultralow breast cancer (5–7). In preclinical models, its effects may involve CTSL-mediated extracellular payload release, FcγR-mediated phagocytosis and DXd-associated TLR4/STING signalling (16). Whether these mechanisms account for variation in patient benefit requires human pharmacodynamic validation. T-DXd can also increase tumour-cell CD47 expression; in HER2-transgenic models, CD47 blockade enhanced antitumour immunity and durable CD8^+^ T-cell memory (16).

### 4.3 TROP2-directed ADCs: payload-associated injury and a separate TROP2 immune-exclusion hypothesis

Dato-DXd and its DXd payload induced CALR exposure, ATP secretion and HMGB1 release *in vitro* and produced a TROP2-dependent bystander effect (44). Separately, Wu et al. showed that a TROP2/claudin programme contributed to immune exclusion and that targeting TROP2 with hRS7, the antibody component of SG, improved anti-programmed cell death protein 1 (PD-1) activity in immunocompetent models (45). That study did not establish the same mechanism for intact SG or Dato-DXd. Dato-DXd showed clinical activity in HR-positive/HER2-negative and TNBC cohorts (46) and antitumour activity in patient-derived breast cancer xenografts (47), but neither study tested whether the intact ADC reverses hRS7-associated immune exclusion. A combined effect of TROP2 targeting and topoisomerase I-inhibitor payloads on immune accessibility therefore remains an author-proposed hypothesis requiring ADC-specific validation.

### 4.4 Fc-mediated antigen handling by antibody scaffolds

The antibody component can influence tumour-immune interactions before payload release. Trastuzumab can promote Fc-receptor-mediated uptake and cross-presentation of HER2 antigen by dendritic cells, increasing expansion of HER2-specific cytotoxic T cells (48). T-DM1 and T-DXd therefore do more than attach a cytotoxic payload to trastuzumab. Their activity may reflect the combined effects of receptor blockade, Fc-mediated antigen uptake and payload-induced cell death. For TROP2-directed ADCs, whether the antibody scaffold retains sufficient Fc activity, which Fcγ receptors are expressed by tumour-associated macrophages, and whether Fc glycosylation alters phagocytosis remain unresolved questions.

These observations motivate construct-specific tests of Fc engineering. In HER2-targeting antibodies, a pH-selective Fc variant showed conditional FcγRIIIa engagement and antibody-dependent cellular cytotoxicity in preclinical systems (49). Whether this design preserves ADC pharmacokinetics, improves phagocytosis or reduces normal-tissue injury remains unknown and requires direct testing in conjugated constructs.

### 4.5 Distinct breast cancer subtypes provide distinct ICD substrates

HER2-positive breast cancer can provide high target abundance and trastuzumab-scaffold activity, but heterogeneity, altered epitopes and truncated HER2 forms may reduce effective binding. Alternative splicing can generate HER2 isoforms lacking antibody-binding domains, illustrating that conventional assay-detected expression does not necessarily equal the abundance of accessible therapeutic epitopes (50). HER2-low disease has broadened HER2-targeted ADC development (51), with early-phase disitamab vedotin data in HER2-overexpressing and HER2-low advanced breast cancer (52). The truncated HER2 isoform p95HER2 can drive an immunosuppressive interleukin-6/programmed death-ligand 1 (IL-6/PD-L1) programme that limits T-DXd activity (53). Treatment-induced CD47 expression is a separate preclinical observation (16).

In a 1,489-patient breast cancer series, luminal A/B HER2-negative tumours had lower tumour-infiltrating lymphocyte densities than TNBC and HER2-positive tumours (54). This population-level contrast does not define the immune state of an individual HR-positive/HER2-negative tumour or establish that endocrine-associated suppression must be reversed to generate immune memory. Biomarker strategies should therefore be matched to the ADC construct, disease context and clinical task.

Brain, liver and bone metastases present distinct pharmacological and immune contexts, but ADC–ICI-specific interactions at these sites remain insufficiently defined. In experimental breast cancer brain metastases, blood–tumour barrier permeability varied markedly across lesions, arguing against a uniform exposure assumption (55). In other tumour types, liver metastases can suppress systemic checkpoint responses through macrophage-mediated elimination of activated T cells (56); this mechanism should not be presumed to operate identically during breast cancer ADC treatment. Organ-specific outcomes and, where feasible, multisite sampling should be reported. **Figure 3** summarises these subtype-specific immune contexts and candidate barriers as context-dependent hypotheses rather than established differences in ICD competence.

**Figure 3:**
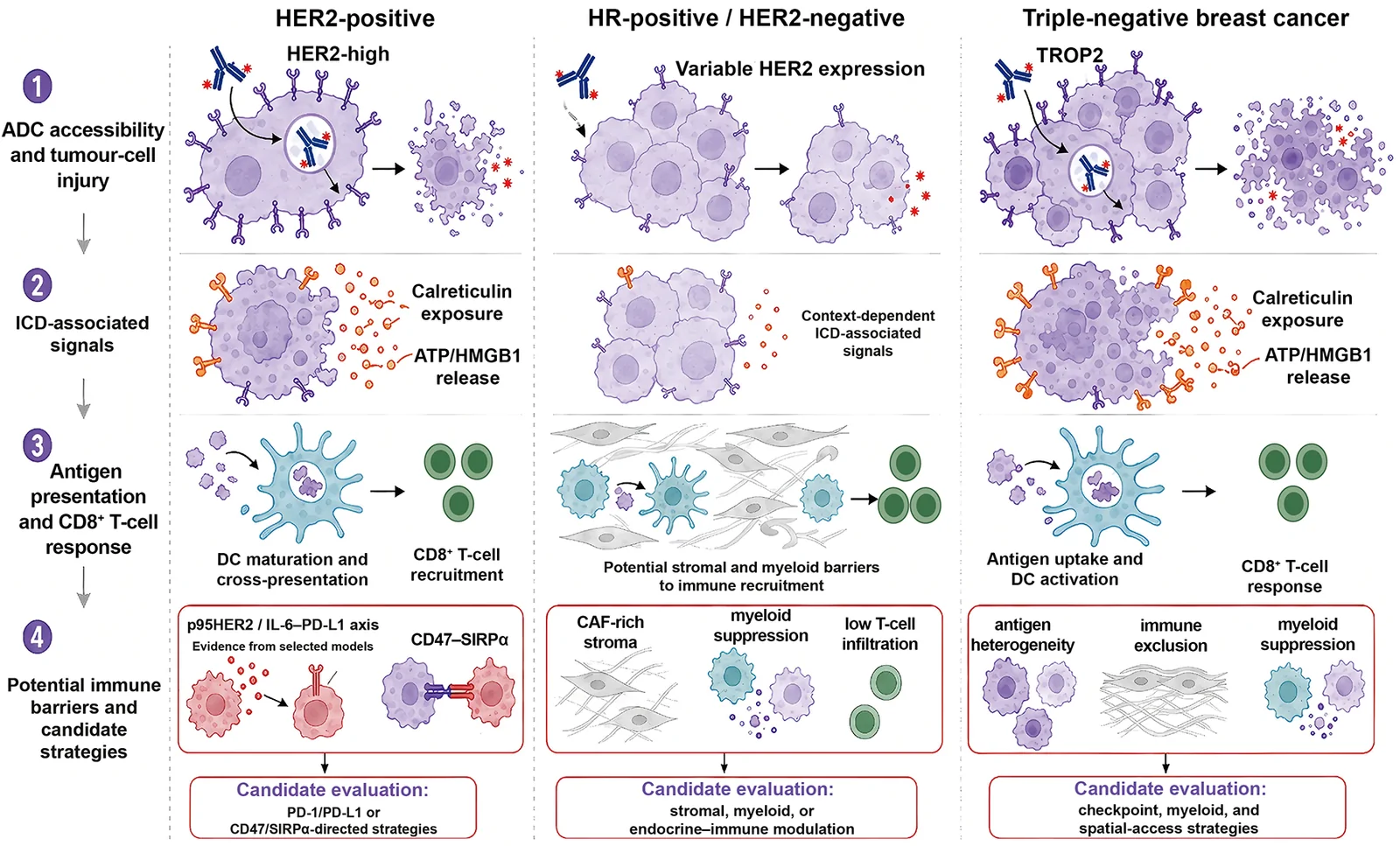
Subtype-specific contexts that may shape ADC-associated immune activity in breast cancer. HER2-positive, HR-positive/HER2-negative and triple-negative breast cancers are compared across ADC accessibility, ICD-associated signals, antigen presentation and candidate immune barriers. The comparisons represent context-dependent hypotheses rather than established differences in ICD competence. The p95HER2/IL-6–PD-L1 axis is an example from selected models. Stromal and myeloid barriers, antigen heterogeneity and immune exclusion are shown as potential constraints, not features exclusive to the subtype in which they are illustrated. Target expression and immune context vary within each subtype; cell numbers and signal densities are schematic rather than quantitative. Candidate combinations are research directions and not treatment recommendations.

## 5 Clinical evidence for ADC–ICI combinations: trial design, efficacy and safety

### 5.1 Current clinical evidence: activity is established, whereas mechanism remains to be demonstrated

Before first-line combination data became available, SG monotherapy had shown clinical activity in refractory metastatic TNBC, and the randomised ASCENT trial established efficacy with subsequent final, safety and subgroup analyses (57–60). ASCENT-04/KEYNOTE-D19 enrolled 443 patients with previously untreated PD-L1-positive advanced TNBC, defined by a combined positive score of at least 10. SG plus pembrolizumab prolonged median progression-free survival compared with chemotherapy plus pembrolizumab (11.2 versus 7.8 months; hazard ratio 0.65, 95% confidence interval 0.51–0.84; P<0.001) (12). Objective response rates were 60% and 53%, and median response durations were 16.5 and 9.2 months, respectively. Overall survival was immature in the primary report. These data demonstrate superiority of the SG–pembrolizumab treatment strategy in the studied population; they do not establish biological synergy or an ICD-mediated mechanism.

The trial compared SG plus pembrolizumab with chemotherapy plus pembrolizumab rather than using a factorial design containing SG and pembrolizumab monotherapy groups. Mechanistic attribution therefore requires embedded pharmacodynamic studies or complementary experimental designs. Paired tumour sampling, TCR tracking and exposure–response analyses could test whether the clinical advantage is accompanied by antigen-specific immune activation, while preserving the distinction between association and mediation.

### 5.2 Clinical activity and interpretive limits of ADC combination trials

In the phase Ib DS8201-A-U105 study, T-DXd plus nivolumab produced confirmed objective responses in previously treated HER2-positive and HER2-low metastatic breast cancer, but the cohort was small and lacked a T-DXd monotherapy control (61). In the phase Ib/II BEGONIA platform, first-line Dato-DXd plus durvalumab showed activity in advanced TNBC (62). T-DXd plus durvalumab was evaluated separately in HR-negative/HER2-low disease, with different eligibility, follow-up and toxicity (63). These nonrandomised cohorts cannot establish relative biological synergy by cross-cohort response-rate comparison. Outside ADC–ICI regimens, T-DXd plus pertuzumab has also undergone randomised phase III evaluation in HER2-positive metastatic breast cancer (64).

ADC–ICI regimens should therefore be interpreted according to the specific construct, disease setting, biomarker selection, prior therapy, comparator and follow-up. In single-arm or nonfactorial studies, response rates demonstrate activity but do not quantify pharmacological or immunological synergy. **Table 2** compares the study populations, treatment arms, endpoints and principal findings of the ADC–ICI trials discussed here, with their design-specific interpretive limits.

**Table 2:** Selected clinical studies of ADC–ICI combinations in breast cancer: design, endpoints, results and interpretive limits.

Study, phase and population	Regimen and comparator	Primary endpoint and key finding	Interpretive boundary
ASCENT-04/KEYNOTE-D19; phase III; first-line PD-L1-positive (CPS ≥10) advanced TNBC; n=443 (12).	SG + pembrolizumab versus chemotherapy + pembrolizumab.	Central-review PFS: 11.2 versus 7.8 months; HR 0.65 (95% CI 0.51–0.84). Overall-survival data were immature.	Randomised evidence for the treatment strategy, not for biological synergy or ICD mediation; no SG-only arm.
DS8201-A-U105; phase Ib; previously treated HER2-positive (n=32) or HER2-low (n=16) metastatic breast cancer (61).	T-DXd + nivolumab; no concurrent control.	Confirmed ORR: 65.6% and 50.0%, respectively. One grade 5 ILD/pneumonitis event occurred in the HER2-positive cohort.	Activity and safety signals only; the incremental effect of nivolumab cannot be estimated.
BEGONIA arms 7/8; phase Ib/II; first-line advanced TNBC; n=62 (unselected PD-L1) and n=33 (PD-L1-high) (62).	Dato-DXd + durvalumab in each arm; no concurrent control.	Safety and confirmed ORR: 79.0% and 81.8%, respectively; median follow-up differed (35.0 versus 10.7 months).	Different selection and follow-up preclude an arm-to-arm efficacy inference; neither arm proves synergy.
BEGONIA arm 6; phase Ib/II; first-line HR-negative/HER2-low advanced breast cancer; n=58 (63).	T-DXd + durvalumab; no concurrent control.	ORR and safety: ORR 62.1% (36/58), below the protocol-specified 66.6% objective; drug-related ILD/pneumonitis 20.7%.	Regimen-specific activity and toxicity; no isolated durvalumab benefit or ICD mechanism can be inferred.
I-SPY2.2; adaptive phase II; high-risk stage II/III breast cancer; n=106 treated in the combination Block A (66).	Dato-DXd + durvalumab in Block A, followed by response-adapted Blocks B/C; no prespecified direct test of the ICI increment.	pCR-based graduation: Block A graduated in the immune-positive subtype; the full sequence graduated in the HR-negative/immune-negative subtype.	A subtype- and sequence-specific signal, not proof of a Dato-DXd–durvalumab interaction or improved event-free survival.
TROPION-Breast03; phase III; residual invasive TNBC after neoadjuvant therapy; planned n≈1,075 (13).	Dato-DXd + durvalumab versus Dato-DXd alone versus investigator’s choice (capecitabine and/or pembrolizumab).	Primary endpoint: invasive disease-free survival. Efficacy results were unavailable at the review cutoff.	The ADC-only arm may help assess the addition of durvalumab; no efficacy or ICD conclusion yet.
ASCENT-05/OptimICE-RD; phase III; residual invasive TNBC after neoadjuvant therapy; planned n=1,514 (14).	SG + pembrolizumab versus pembrolizumab with or without capecitabine.	Primary endpoint: invasive disease-free survival. Efficacy results were unavailable at the review cutoff.	Will test a postoperative treatment strategy; without an SG-only arm, it cannot isolate biological synergy.

Data cutoff: 31 July 2026. Planned n is shown for ongoing trials; reported n is cohort-specific. Nonrandomised ORRs are not cross-trial comparisons. CPS, combined positive score; ILD, interstitial lung disease; ORR, objective response rate; pCR, pathological complete response; PFS, progression-free survival.

### 5.3 Evaluation in earlier-stage disease

Earlier-stage disease offers opportunities for paired tissue sampling and evaluation of recurrence-related endpoints, but it should not be assumed to possess a uniformly more competent immune system. In I-SPY2.2, Dato-DXd did not meet the prespecified Block A graduation threshold in any subtype; the complete sequential treatment strategy graduated in the HER2-negative, immune-negative, DNA-repair-deficiency-negative response-predictive subtype (65). The same platform separately evaluated a Dato-DXd–durvalumab strategy in early-stage breast cancer (66). TROPION-Breast03 and ASCENT-05/OptimICE-RD are ongoing phase III studies of postoperative strategies in TNBC and had no reported efficacy results at the review cutoff (13, 14). DESTINY-Breast11 evaluated neoadjuvant T-DXd-based strategies in high-risk HER2-positive early breast cancer (67). In curative settings, potential gains must be assessed alongside pulmonary, haematological, reproductive and immune toxicities.

### 5.4 Interpreting efficacy: distinguish alternative cytotoxic benefit from immune synergy

An improved response with an ADC–ICI regimen may reflect stronger ADC cytotoxicity, longer tumour exposure, patient selection or an immune interaction. Randomised comparisons against a relevant standard establish the effect of a treatment strategy, whereas a prespecified interaction or additivity design is needed to evaluate synergy. Paired specimens can demonstrate pharmacodynamic changes but cannot, on their own, prove that ICD mediates clinical benefit. Mechanistic claims should therefore be matched to the comparator and biological controls used in each study.

Depth of response is not the only endpoint of interest. If ADC-induced immune priming generates durable memory, it may be reflected in longer response duration, disease control after treatment withdrawal, altered sensitivity to subsequent therapy or fewer new metastases. The longer remission observed with the combination in ASCENT-04 warrants attention (12), but the contribution of immune mechanisms remains to be assessed using paired pharmacodynamic and clonal analyses. In early-stage disease, pathological complete response is a useful early endpoint but cannot substitute for event-free survival; residual-lesion immune composition, ctDNA status and tissue-resident clonal T cells may more closely reflect recurrence risk.

### 5.5 Safety and agent-specific toxicity constraints

T-DXd-associated interstitial lung disease (ILD)/pneumonitis may involve drug disposition, tissue susceptibility and inflammation, but an agent-specific causal relationship with excessive circulating free payload has not been established. The reported 15.4% pooled incidence came from 1,150 patients in nine phase I/II T-DXd monotherapy studies across several tumour types; breast cancer represented 44.3% of that population (68). It is not an estimate for breast cancer ADC–ICI combinations. Published multidisciplinary guidance recommends permanent T-DXd discontinuation for grade 2 or higher ILD/pneumonitis (69), consistent with current US prescribing information, and therefore limits discussion of rechallenge.

Combination trials should prospectively capture baseline pulmonary risk, symptoms, oxygenation, imaging findings, treatment exposure and adjudicated toxicity. Bronchoalveolar lavage may be appropriate in selected diagnostic settings but is not a routine biomarker procedure. The objective is to define a regimen-specific therapeutic window that balances tumour control against irreversible pulmonary, cardiac, haematopoietic and reproductive harm, particularly in curative settings.

### 5.6 Dynamic monitoring may be more informative than single-antigen testing

Baseline HER2 or TROP2 measurements describe target status but may not capture payload sensitivity or evolving immune responses. A prospective 2026 circulating tumour cell (CTC) study included 35 patients and found that a decline exceeding 80% at approximately three weeks was associated with more durable benefit (70). This exploratory threshold requires independent validation before clinical use. In an exploratory phase I dataset of 151 T-DXd-treated patients, continuous quantitative HER2 image analysis stratified progression-free survival more clearly than conventional categorical scoring (71). This approach likewise requires independent prospective validation and should not replace standard HER2 testing.

Clinical monitoring can be considered at three levels: readily implementable measures such as CTCs, ctDNA, complete blood counts and imaging; spatial assessment of antigens, cellular injury, DAMPs and immune cells in paired tissues; and research assays such as TCR sequencing, single-cell profiling and circulating proteomics. Their incremental predictive value, feasibility and sampling failure rates should be compared prospectively rather than assuming that more complex profiling is superior.

## 6 Resistance checkpoints across the ADC–ICD immune cascade

We propose a four-stage qualitative framework to organise potential failure points in the conversion of ADC-associated tumour-cell injury into adaptive immunity. The stages are heuristic and may interact nonlinearly; the framework does not assert a validated temporal sequence or mathematical relationship.

Stage 1, ignition: the ADC must reach the tumour and deliver sufficient payload. Antigen density and heterogeneity, tumour perfusion, interstitial pressure, endocytic and lysosomal function, proteases such as CTSL, and drug-efflux pumps jointly determine effective exposure.

Stage 2, signal transmission: tumour-cell injury must generate a coordinated set of antigenic and adjuvant signals. Cell-death morphology alone does not determine immunogenicity. Ineffective transmission may arise from insufficient or mistimed CALR exposure, ATP/HMGB1 release, type I interferon signalling, antigen availability or innate sensing.

Stage 3, immune reception: cDC1 and other cross-presenting dendritic cells must complete antigen uptake, maturation, migration and cross-presentation before expanding tumour-reactive CD8^+^ T-cell clones. Tumour-derived extracellular vesicles in triple-negative breast cancer can deliver cell division cycle 37 (CDC37) to dendritic cells, impairing antigen transport from endosomes to the cytosol and weakening cross-presentation. Antigen release therefore does not guarantee that the immune system can receive the signal (72).

Stage 4, adaptive braking: tumours can induce feedback inhibition through PD-L1, CD47, transforming growth factor-β (TGF-β), IL-6, adenosine, myeloid-derived suppressor cells (MDSCs), regulatory T cells (Tregs) and dysfunctional T cells. p95HER2 can drive immunosuppressive programmes involving PD-L1 and IL-6 and limit T-DXd activity (53), whereas TROP2/claudin organisation can promote spatial immune exclusion (45). A breast-cancer cell-line study linked DXd-based ADC exposure to senescence and a senescence-associated secretory phenotype (73); whether this alters immune clearance or patient outcomes remains untested.

The framework proposes that weakness at any stage may limit immune conversion, but biological compensation and nonlinear interactions are possible. It should therefore be used to organise testable hypotheses rather than to infer that any component has a multiplicative effect or that a single near-zero measurement predicts treatment failure.

### 6.1 Target loss and impaired intracellular payload delivery

Failure of ignition can result from low antigen abundance or from barriers affecting antigen accessibility, epitope structure or intracellular drug delivery. Alternative HER2 splicing can generate isoforms lacking antibody-binding domains (50), and acquired HER2 loss or binding-interface mutations have been linked to T-DXd resistance (74). Anti-HER2 antibody and ADC imaging studies show that binding and internalisation can constrain penetration beyond perivascular regions (18, 19). For TROP2-directed ADCs, antigen heterogeneity, tight-junction programmes and payload sensitivity remain candidate contributors to primary resistance rather than validated predictors (45). The ignition domain should therefore capture binding epitopes, spatial heterogeneity, drug penetration and construct-relevant linker cleavage rather than treat an immunohistochemistry score as a complete surrogate.

### 6.2 Inadequate danger signalling and defective antigen presentation

Similar amounts of tumour-cell death can produce different immune outcomes (23). Immunogenicity cannot be inferred from rapid membrane disruption or another morphological category alone (23). Relevant variables include CALR exposure, autophagy-dependent ATP release and the redox state of HMGB1 (23, 26, 34). Type I interferon signalling, antigen availability, dendritic-cell competence and the surrounding suppressive milieu also shape the immune response (23). In HER2-positive breast cancer cell models, T-DXd induced sustained senescence and a senescence-associated secretory phenotype (SASP) (73). Whether this response recruits suppressive myeloid cells or impairs ADC-associated immune priming requires functional testing in immunocompetent models. Within the proposed framework, transmission failure can therefore reflect insufficient danger signalling or a tissue response that favours tolerance or suppression (23, 34).

### 6.3 T-cell dysfunction and myeloid suppression

ICIs primarily release inhibitory signalling in existing T-cell responses and cannot replace antigen uptake or initial priming. Defective dendritic-cell recruitment or cross-presentation, including the CDC37-associated mechanism described in TNBC models (72), can limit priming. By contrast, tumour-cell loss of MHC class I or β2-microglobulin disrupts antigen presentation to CD8^+^ T cells, as shown by B2M deletion in TNBC co-cultures (75) and by antigen-presentation defects in clinical checkpoint-resistant tumours outside breast cancer (76). Dendritic cells can nevertheless cross-present antigens from MHC-I-negative transformed cells in an experimental system (77). These are distinct resistance checkpoints; the CDC37 finding remains preclinical and has not been established as a clinical ADC-resistance mechanism.

### 6.4 Adaptive immune escape and treatment-induced senescence

PD-L1 is one of several adaptive brakes. Treatment-associated changes in CD47, IL-6, TGF-β, adenosine, myeloid-derived suppressor cells (MDSCs), regulatory T cells (Tregs), T-cell exhaustion and antigen-presentation pathways may emerge at different times. Evaluating CD47/signal regulatory protein α (SIRPα), PD-1/PD-L1, senescence-directed or myeloid-directed combinations according to these changes is a research strategy, not a clinically validated treatment sequence. Each proposed combination requires evidence of pathway dependence and regimen-specific safety. **Figure 4** summarises the author-proposed framework and its candidate resistance breakpoints.

**Figure 4:**
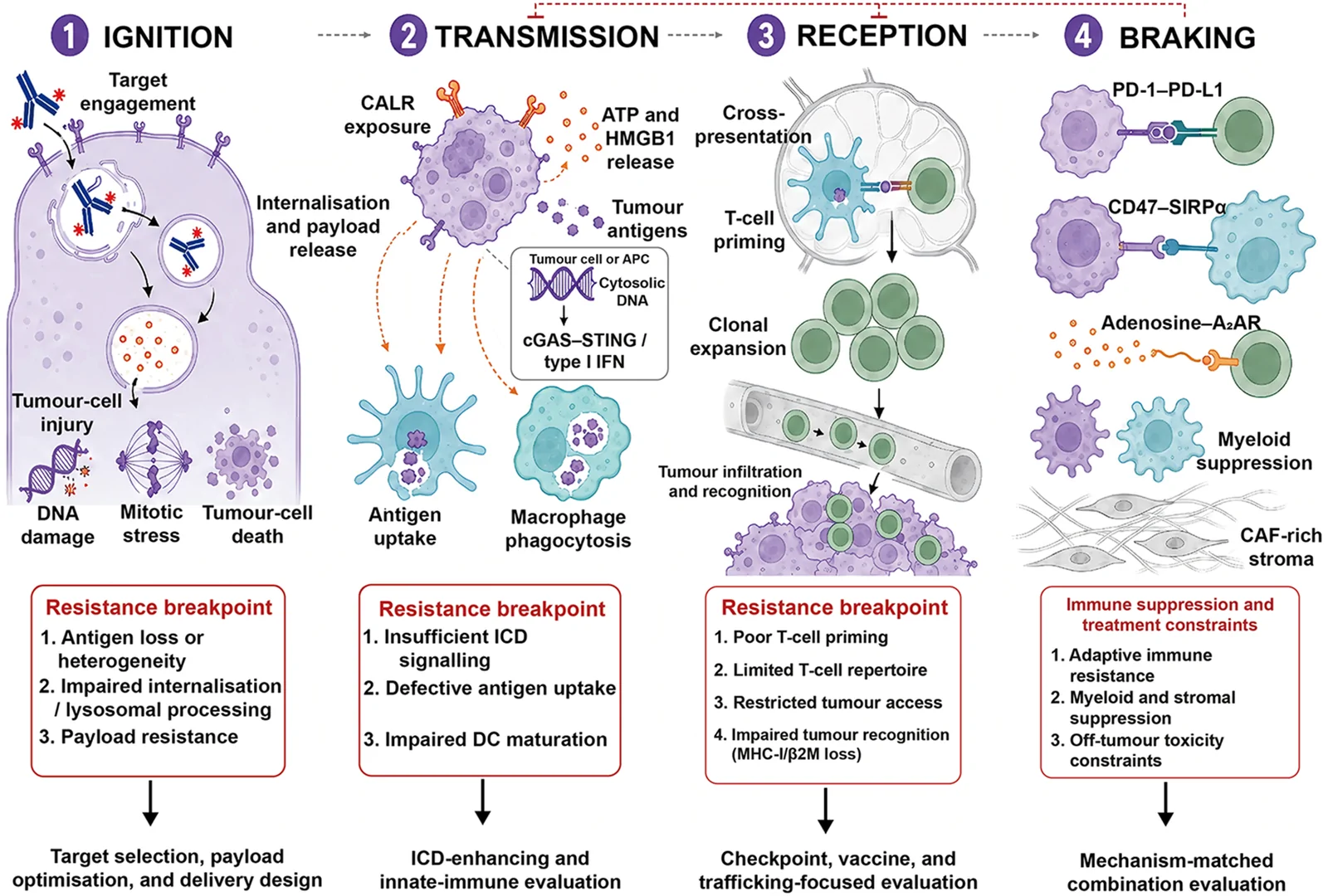
Author-proposed four-stage framework for potential failure points in ADC-associated immune conversion. Ignition comprises target engagement, payload delivery and initial tumour-cell injury; transmission comprises antigen availability, uptake, phagocytic handling and innate danger signalling; reception comprises dendritic-cell processing, T-cell priming, trafficking and tumour recognition; and braking comprises checkpoint, myeloid, adenosine and stromal constraints. Tumour-cell death in the ignition domain does not by itself establish functional ICD. Dendritic-cell cross-presentation and tumour-cell recognition are distinct checkpoints; loss of tumour-cell MHC class I or β2-microglobulin (β2M) is shown as a potential barrier to the latter. Off-tumour toxicity is shown as a treatment constraint. Dashed red lines ending in T-bars indicate inhibitory effects across domains. The domains may interact nonlinearly. Grey dashed arrows organise the framework without specifying a validated temporal sequence or a multiplicative relationship. Intervention labels denote hypotheses for prospective evaluation.

## 7 Biomarker development and clinical validation

To translate these concepts into testable biomarker studies, we propose a qualitative ADC–ICD competence framework. Its four domains are ADC accessibility (A), danger signalling (D), immune reception (R) and immune braking (B). The domains organise biological questions rather than form a validated biomarker index.

No numerical equation, weighting scheme or clinical threshold is assigned. The intended clinical task must be specified before model development because prediction of ADC monotherapy response, incremental ADC–ICI benefit, early pharmacodynamic activity and resistance at progression require different variables and study designs. **Table 3** summarises candidate measures and their limitations.

**Table 3:** Four domains of the proposed ADC–ICD competence framework and candidate measurements.

Domain	Candidate measures by sampling stage	Suggested methods	Biological interpretation and limitations
A. ADC accessibility	Pretreatment target abundance, accessible epitopes, spatial heterogeneity, perfusion and internalisation; CTSL only for relevant linker chemistry	Digital pathology, multiplex imaging, spatial proteomics and construct-specific imaging or pharmacokinetics	Describes delivery opportunity; does not measure payload sensitivity or immune conversion
D. danger signalling	Early on-treatment CALR exposure, ATP/HMGB1 release, p-eIF2α and cGAS–STING/type I interferon activity; γH2AX as a DNA-damage marker	Paired tissue assays, standardised biofluids, transcriptomics and proteomics	Indicates cellular stress and immunogenic potential; does not independently establish functional ICD
R. immune reception	cDC1 abundance and activation, cross-presentation, TCR clonal expansion, CD8^+^ T-cell infiltration and tumour-cell MHC-I integrity	Multiplex imaging, functional cross-presentation assays, single-cell profiling and TCR sequencing	Separates dendritic-cell priming from subsequent tumour-cell recognition and killing
B. immune braking	Dynamic PD-L1, CD47, adenosine-axis, MDSC/Treg, stromal and senescence-associated features	Multiplex pathology, plasma proteins and spatial or single-cell profiling	Describes candidate resistance states; no marker currently defines a validated treatment sequence

The framework distinguishes pretreatment characteristics from on-treatment pharmacodynamic changes and progression-associated resistance. Baseline sampling may characterise target accessibility and immune context; early on-treatment sampling can assess cellular injury, danger signalling and immune engagement; and progression sampling can identify acquired resistance. The proposed 24–72 h interval is a hypothesis for window studies and requires agent-specific pharmacokinetic and pharmacodynamic validation.

### 7.1 Intended use and domain-specific measurements

Candidate measures should be selected according to the intended use. Accessibility may include target intensity, accessible epitopes, spatial heterogeneity, perfusion and internalisation; CTSL is relevant only to constructs whose linker biology supports extracellular cleavage. Danger signalling may include treatment-induced CALR exposure, ATP/HMGB1 release and type I interferon-related activity. γH2AX should be interpreted as a DNA-damage-response marker rather than a released danger signal. Immune reception may include cDC1 abundance, dendritic-cell cross-presentation, TCR clonal expansion and CD8^+^ T-cell infiltration, while tumour-cell MHC-I integrity should be considered separately as a determinant of effector recognition. Immune braking may include checkpoint, adenosine, myeloid, Treg, stromal and senescence-associated features.

A future quantitative model would require one prespecified clinical task. Variables that predict ADC monotherapy response need not predict incremental benefit from adding an ICI, and early pharmacodynamic markers differ from recurrence or resistance markers. The present framework therefore does not propose a universal score. Quantification should be attempted only after the outcome, sampling time, candidate variables and modelling strategy have been prespecified.

Model fitting and feature selection should occur within development data, with internal validation to estimate optimism (78). A locked model should then be evaluated in independent data for discrimination, calibration and, if it will guide treatment, clinical utility (78). Thresholds should be fixed before external validation rather than repeatedly optimised in that cohort. Predicting incremental ADC–ICI benefit additionally requires a treatment-by-biomarker interaction analysis in randomised data or another design that can estimate differential treatment effect.

### 7.2 Candidate biomarker domains for ADC–ICI combinations

Dynamic biomarkers are vulnerable to pre-analytical variation. Ischaemia time, fixation, biopsy site, necrosis and concomitant treatment can alter protein and RNA measurements, while ATP and HMGB1 are affected by sample handling and systemic inflammation. Trials should standardise dose-to-biopsy intervals and processing, sample the same lesion when feasible, and distinguish tumour nests, stroma and necrotic regions. Tissue, plasma and peripheral-blood mononuclear cells provide complementary rather than interchangeable information.

High-dimensional profiling should be reduced to reproducible assays only after analytical and clinical validation. A simplified panel cannot be assumed to preserve the performance of the discovery platform and requires its own validation. **Figure 5** illustrates dynamic sampling, the four-domain framework and a staged validation pathway; it does not define a score or treatment algorithm.

**Figure 5:**
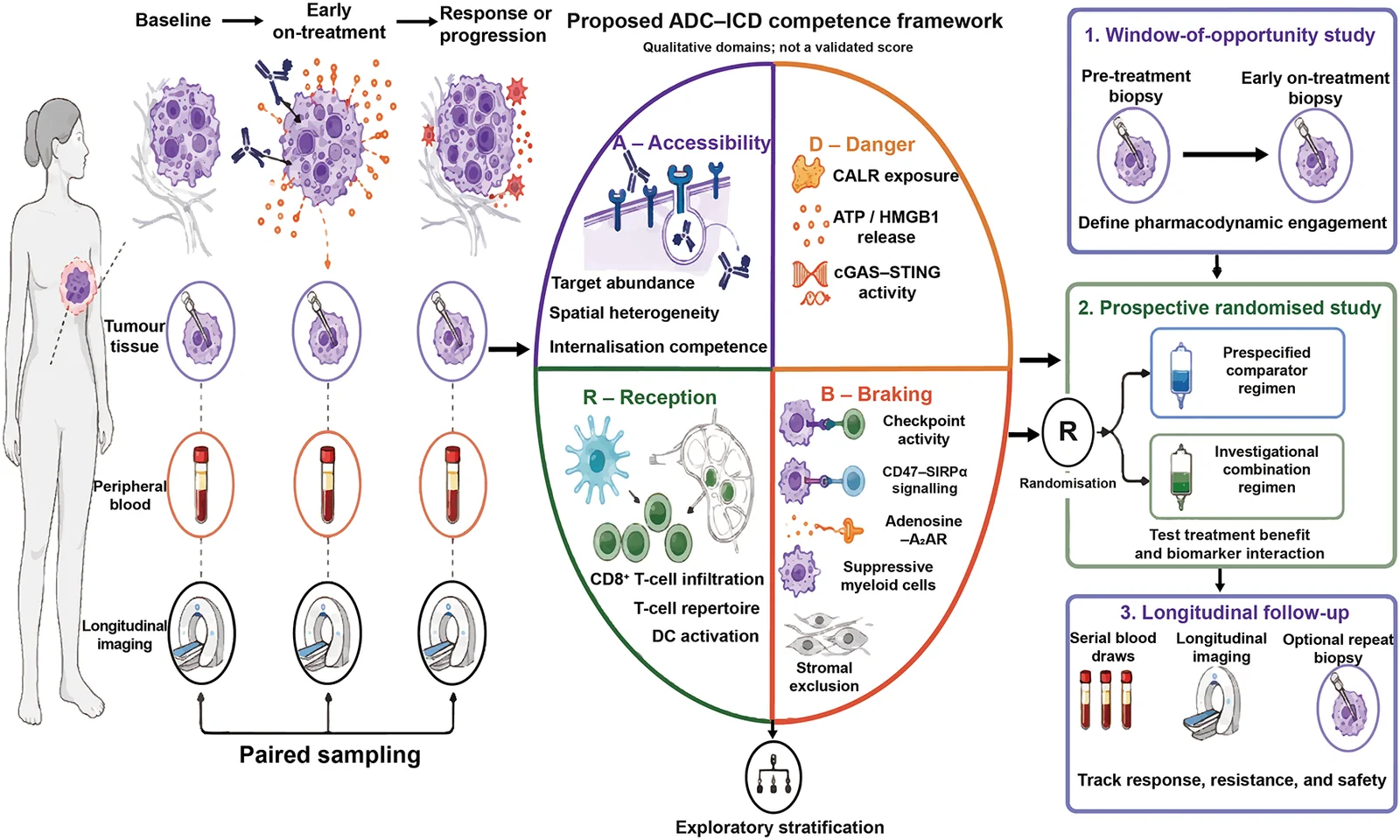
Dynamic sampling and prospective evaluation of the proposed ADC–ICD competence framework. Serial tissue, blood and imaging assessments are shown at baseline, early treatment and response or progression. Accessibility **(A)**, danger signalling **(D)**, immune reception (R) and immune braking **(B)** are qualitative domains, not components of a numerical score. Pretreatment predictors, on-treatment pharmacodynamic markers and progression-associated resistance markers should be analysed separately. Window studies assess pharmacodynamic engagement. Randomised comparisons of a prespecified comparator regimen and an investigational combination are proposed to test treatment benefit and treatment-by-biomarker interaction. The R in the treatment-allocation symbol denotes randomisation, distinct from the immune-reception domain. Longitudinal follow-up tracks response, resistance and safety. This research pathway does not constitute a validated clinical algorithm.

## 8 Translational research priorities

### 8.1 Test immune mediation with appropriate comparators

A preoperative window study can test whether a defined ADC dose or schedule alters tumour injury and immune readouts relative to an appropriate control. To investigate mediation, subsequent studies should connect treatment, antigen-specific immune change and outcome in the correct temporal order, while addressing competing direct cytotoxic effects and mediator–outcome confounding. Randomised efficacy comparisons answer a different question: whether the treatment strategy improves clinical outcomes. “Synergy” should be reserved for studies with a prespecified interaction or additivity framework.

### 8.2 Prioritise combinations by the measured resistance mechanism

Candidate combinations should be advanced only when the proposed resistance pathway is demonstrated in the relevant construct and model. CD47/SIRPα blockade requires evidence that phagocytic inhibition limits antigen uptake; checkpoint blockade requires a primed but inhibited T-cell response; and myeloid, adenosine or senescence-directed agents require corresponding functional evidence. These are research priorities, not a validated clinical sequence, and each combination needs an explicit toxicity assessment.

### 8.3 Next-generation ADC design hypotheses

Dual-payload ADCs have been developed to address tumour heterogeneity and resistance in preclinical breast cancer models (79). A distinct B7-H3-directed construct carrying monomethyl auristatin F (MMAF) and a Toll-like receptor 7/8 (TLR7/8) agonist increased cytotoxic and immune activity in a TNBC model (80). Whether similar immune payloads, STING-agonist conjugates, bispecific or conditionally activated ADCs improve patient outcomes remains untested. Their evaluation should measure local immune activation alongside systemic cytokine, hepatic and myeloid toxicity.

### 8.4 Match endpoints to the level of biological inference

A trial intended to measure pharmacodynamic engagement should prespecify tissue timing, cellular compartment and assay reproducibility. A trial intended to test immune dependence needs functional controls or a design that can separate immune effects from direct cytotoxicity. Clinical durability requires its own endpoints, such as response duration or event-free survival; neither prolonged control nor a T-cell repertoire change alone establishes immune memory or ICD causation.

### 8.5 Develop treatment-selection biomarkers separately from companion diagnostics

The proposed A–D–R–B domains should first be evaluated for a prespecified task, such as predicting benefit from adding an ICI to an ADC. Analytically validated baseline assays are suitable candidate treatment-selection variables; on-treatment changes instead measure pharmacodynamic activity and should not be substituted for pretreatment predictors.

A randomised comparison with a relevant control is needed to evaluate whether a baseline marker predicts differential treatment benefit, rather than simply identifying patients with a better prognosis. Biomarker cutoffs, interaction analyses, missing-sample handling and the clinical decision to be supported should be prespecified. Serial blood and optional repeat biopsies can then assess resistance, safety and assay feasibility without redefining the locked prediction rule.

A companion diagnostic is an assay considered essential for the safe and effective use of a corresponding therapeutic product, including selection, toxicity-risk assessment or monitoring (81). A marker associated with incremental ADC–ICI benefit is a candidate predictive biomarker of differential treatment effect, not automatically a companion diagnostic. Regulatory qualification would require a specific intended use and separate analytical and clinical validation.

### 8.6 ADC sequencing and payload cross-resistance

As HER2- and TROP2-directed ADCs enter the same treatment pathway, patients may receive two topoisomerase I-inhibitor ADCs sequentially. In the retrospective ADC-Low study of 179 patients, median progression-free survival on the second ADC was 2.7 months, and 54.4% had primary resistance (82). Other observational series and a systematic review also report limited activity of some second ADCs (83, 84). These later-line outcomes raise concern about cross-resistance, but treatment order, additional therapies and clonal evolution preclude a causal attribution. By contrast, acquired topoisomerase I (TOP1) mutations detected after ADC exposure experimentally conferred resistance to both SN-38 and deruxtecan in a subset of cases (85). Payload cross-resistance is therefore a demonstrated mechanism in some tumours, not a universal explanation for sequential failure. Sequencing studies should distinguish target loss or altered binding (74) from changes in payload sensitivity, drug efflux and DNA repair. Payload diversification has preclinical support (86), and DNA-repair inhibition has been studied as a resistance strategy (87), but neither is an established clinical sequencing rule. Prospective cohorts with progression biopsies, ctDNA, exposure data and explicit prior-therapy histories are needed to estimate mechanism-specific benefit.

## 9 Limitations and evidence gaps

The main limitation is the uneven maturity of evidence across ADC constructs: cellular hallmarks are common, but functional immune-dependence studies and paired human mechanistic data are sparse. The following sections address model validity, causal inference and biomarker feasibility.

### 9.1 Model systems and causal inference remain key limitations

Human tumour-cell xenografts lack a complete human immune system, syngeneic models differ in antigen expression and ADC cross-reactivity, and humanised models incompletely reproduce myeloid and lymph-node biology. Organoid–immune co-cultures resolve selected cell interactions but not systemic pharmacology or organ toxicity. Vaccination/rechallenge and immune-dependence experiments in immunocompetent systems remain strong functional tests within those models; the limitation is that no single model establishes relevance in patients. Convergent evidence across cellular systems, immunocompetent models and paired human specimens is therefore required.

Associations in clinical samples do not establish causality. Increased CD8^+^ T-cell density after treatment may reflect immune recruitment or a relative change after tumour shrinkage, and circulating HMGB1 may derive from tumour or normal-tissue injury. Mediation analysis can test whether observations are consistent with a treatment-to-immune-response-to-outcome pathway, but causal interpretation requires strong assumptions about confounding, measurement and temporal ordering.

### 9.2 Research hotspots may also promote conceptual homogenisation

Conceptual homogenisation is another risk. Some studies apply CALR, ATP and HMGB1 assays to new payloads without testing antigen-specific immunity, while clinical development often defaults to PD-1/PD-L1 combinations despite alternative bottlenecks in phagocytosis, dendritic-cell function, adenosine signalling or myeloid biology. The relevant question is which specific limiting step a construct or combination addresses and which control can falsify that mechanism.

### 9.3 Feasibility, equity of sampling and cost

Spatial and multi-omic platforms may improve mechanistic resolution but can restrict participation to centres and patients able to provide large, high-quality specimens. Advanced breast cancer is often represented by small core biopsies, decalcified bone specimens or archival tissue. Discovery and simplified assays should therefore be developed as separate products: any simplified version based on routine pathology, blood or imaging requires independent analytical and clinical validation rather than assumed equivalence to the discovery platform.

Genetic background, prior therapy, pulmonary disease, organ involvement and access to repeated sampling may alter efficacy, toxicity and model performance. Development and validation cohorts should represent clinically relevant variation, while sampling failures and missing data should be reported. Otherwise, biomarker frameworks may apply only to selected populations able to undergo repeated tissue profiling.

## 10 Conclusions

ADCs can influence tumour-cell injury, myeloid phagocytosis, antigen processing and T-cell responses, but the strength and sequence of these effects are agent and context specific. T-DM1, T-DXd and TROP2-directed ADCs provide different levels of ICD-associated evidence, ranging from molecular hallmarks to immune engagement in preclinical systems. ASCENT-04/KEYNOTE-D19 establishes SG plus pembrolizumab as an effective first-line strategy in PD-L1-positive advanced TNBC without demonstrating that the benefit is mediated by ICD. The proposed accessibility, danger, reception and braking framework offers a qualitative structure for biomarker development rather than a validated score. Progress will depend on construct-specific functional experiments, paired pharmacodynamic studies, randomised treatment-effect analyses and explicit separation of immune association from causal mechanism.
